# Valproate-related neutropenia and lithium-related leukocytosis in patients treated with clozapine: a retrospective cohort study

**DOI:** 10.1186/s12888-023-04659-2

**Published:** 2023-03-15

**Authors:** Chia-Chun Yang, Xi-Yu Wang, Po-Han Chou, Ching-Hua Lin

**Affiliations:** 1 Department of General Psychiatry, Taoyuan Psychiatric Center, No. 71, Longshou St., Taoyuan Dist, 33058 Taoyuan City, Taiwan; 2 Department of Psychiatry, China Medical University Hsinchu Hospital, Hsinchu, Taiwan; 3 Kaohsiung Municipal Kai-Syuan Psychiatric Hospital, Kaohsiung, Taiwan; 4 Department of Psychiatry, School of Medicine, College of Medicine, Kaohsiung Medical University, Kaohsiung, Taiwan

**Keywords:** Clozapine, Valproate, Lithium, Neutropenia, Leukocytosis

## Abstract

**Background:**

Neutropenia is a noteworthy side effect of clozapine, which might warrant this drugs’ discontinuance for safety. Studies have revealed that the risk of neutropenia increases with concurrent administration of valproate, but the evidence was limited. Conversely, lithium may have an ameliorating effect on clozapine-induced neutropenia. This study explored the effects of valproate and lithium on white blood cell counts in patients treated with clozapine.

**Methods:**

We retrospectively investigated the electronic medical records from one tertiary psychiatric hospital in Taiwan and enrolled patients discharged between January 1, 2006, and December 31, 2017, with clozapine prescriptions. We scrutinized their demographic data, medications, and hematological results at discharge and during follow-up outpatient clinic visits over the subsequent 3 years. Patients were classified into four groups: clozapine only (CLO), clozapine and valproate (CLO + VAL), clozapine and lithium (CLO + Li), and clozapine, valproate, and lithium (CLO + VAL + Li). We also identified hematological events (neutropenia or leukocytosis) of these patients during outpatient follow-ups.

**Results:**

Of the included 1084 patients, 55(5.1%) developed neutropenia. Concurrent valproate use (odds ratio [OR] = 3.49) and older age (*p* = .007) were identified as risk factors. Moreover, 453 (41.79%) patients developed leukocytosis. Younger age; male sex; and concurrent use of lithium (OR = 3.39, *p* < .001), clozapine daily dosage, and benzodiazepines were the risk factors for leukocytosis.

**Conclusion:**

Concurrent valproate use and older age are associated with the development of neutropenia in patients treated with clozapine. Concurrent lithium usage, younger age, male sex, and concurrent benzodiazepine use might be related to leukocytosis.

**Supplementary Information:**

The online version contains supplementary material available at 10.1186/s12888-023-04659-2.

## Introduction

Clozapine, a second-generation antipsychotic (SGA), is the most effective antipsychotic for treatment-refractory schizophrenia (TRS) [1, 2], especially in reducing positive psychotic symptoms [1]. Approximately 30% of patients with schizophrenia respond poorly to antipsychotics and are thus clinically categorized as TRS [3]. Clozapine is well-known for its neutropenic side effects. Among the clozapine-induced adverse drug reactions, neutropenia is particularly dangerous because it may eventually develop into agranulocytosis, a potentially life-threatening condition due to high risk of infection [4]. The prevalence of neutropenia (absolute neutrophil count [ANC] < 1500/mL or white blood count [WBC] < 3000) and agranulocytosis (ANC < 500/mL) caused by clozapine was determined to be 2–3% and 0.4%-0.7%, respectively [5–7]. According to recent research, the majority of cases of neutropenia do not deteriorate into agranulocytosis [8]. However, regular blood monitoring may facilitate the early detection of the decreased WBC, enabling prompt management of neutropenia, thereby reducing the risk of agranulocytosis and other complications [5, 9, 10]. Leukocytosis is another possible side effect of clozapine. However, because clozapine-associated leukocytosis tends to be benign and asymptomatic, generally no change in clozapine dosage is needed under accurate clinical monitoring [11].

Clozapine has been augmented with mood stabilizers, including sodium valproate and lithium, to improve the treatment effect for concurrent mood symptoms in schizophrenia and schizoaffective disorder. [12]. The combination of valproate and clozapine was more effective than clozapine monotherapy in alleviating symptoms such as anxiety, depression, and hostility [13]. Likewise, adjunctive lithium to clozapine treatment demonstrated somewhat enhanced efficacy compared with clozapine monotherapy according to the global assessment of functioning scale and positive and negative syndrome scale ratings [14]. However, the concurrent use of clozapine with mood stabilizers has also been associated with an increased risk of blood dyscrasias [15, 16].

The concurrent use of valproate with clozapine may lead to an increased risk of neutropenia. A recent case–control study in the United Kingdom reported that the risk of clozapine-associated neutropenia increased with concurrent sodium valproate use. In this study, the effect of sodium valproate on neutropenia risk was dose-dependent [16]. Sodium valproate per se was reported to induce neutropenia, and the effect of sodium valproate on neutropenia risk was shown to be dose-dependent in some studies [16, 17]. The putative mechanism was that oxidative stress in neutrophils induced by clozapine was intensified by valproate-related reduced glutathione production, resulting in further toxicity to neutrophils and cell apoptosis [16, 18].

Although sodium valproate increases the neutropenia risk, another mood stabilizer, lithium, may increase the risk of leukocytosis when prescribed alone [16, 17, 19, 20]. Therefore, with the leukocytosis effect, lithium was proposed to have efficacy for treating clozapine-associated neutropenia [21]. Lithium may be protective against blood dyscrasias due to its protective effects of progenitor cells, stimulation of G-CSF production and release, and independent stimulation of progenitor proliferation [22].

Observational studies have provided some evidence of the relationships of clozapine with valproate and lithium [15, 16, 18, 21]. The presence of neutropenia is prone to infection and subsequent sepsis [23]. However, data concerning the development of hematological events such as neutropenia or leukocytosis linked to the coadministration of either valproate or lithium are limited. In addition, hematological events related to combining clozapine with both valproate and lithium at the same time is yet to be determined. Therefore, this study aims to explore the relationships between clozapine and concomitant use of valproate, lithium or both valproate and lithium, to identify the clozapine-induced hematological events (i.e., neutropenia and leukocytosis) and determine which concurrent medications may increase the risk of said hematological events. We also analyzed the demographic characteristics and concomitant medications of patients treated with clozapine to determine the factors that may predispose patients to neutropenia or leukocytosis events.

## Materials and methods

### Participants and study design

A retrospective study was conducted at the Taoyuan Psychiatric Center, which is a 957-bed, tertiary psychiatric hospital in northern Taiwan. Patients were enrolled if they had received clozapine at discharge, during the period between January 1, 2006, and December 31, 2017. Enrolled patients were followed up at outpatient clinics for 3 years after discharge (or until December 21, 2020), with regular blood examinations to detect hematological events. We reviewed each patient’s age, sex, medication regimen, and blood data at discharge and subsequent outpatient clinic visits. We also documented each patient’s daily dose of clozapine, concomitant psychotropic medications (e.g., FGAs, SGAs, mood stabilizers, antidepressants, and anxiolytics) and other concurrent medications (e.g., antihypertensives, antidiabetics, antihyperlipidemics, and antiepileptics).

The literature revealed that concurrent selective serotonin reuptake inhibitor (SSRI) therapy reduced the risk of neutropenia [16]; therefore, we also identified different classes of antidepressants, to explore whether there was a similar finding. For each antidepressant, we calculated the defined daily dose (DDD) using data from the World Health Organization Collaborating Centre for Drug Statistics Methodology, to allow a better comparison of different drug classes [24].

The flowchart of patient classification and hematological event identification process is depicted in Supplementary Fig. 1. We classified patients into four groups on the basis of their drug regimen at discharge: clozapine only (CLO), concomitant use of clozapine and valproate (CLO + VAL), concomitant use of clozapine and lithium (CLO + Li), and concomitant use of clozapine, valproate, and lithium (CLO + VAL + Li). In the present study, neutropenia was defined as WBC counts < 3000 or ANC < 1500, and leukocytosis was defined as WBC counts > 11,000. During the subsequent outpatient clinic follow-ups, if any neutropenia or leukocytosis event occurred, we scrutinized the patient’s drug regimen preceding the event. We included those patients with clozapine prescription along with hematological events, confirmed by blood data, in line with the criteria in Naranjo algorithm [25]. For those who experienced more than one episode of neutropenia or leukocytosis during the 3-year observation, only the first episode of neutropenia or leukocytosis was considered in the analysis.

This study protocol was approved by the Taoyuan Psychiatric Center Institutional Review Boards and was conducted in accordance with both the Human Subjects Research Act of Taiwan and the Declaration of Helsinki (2013). Informed consent was not required as all patient data were depersonalized prior to analysis to preserve anonymity.

### Statistical analysis

Analyses were conducted using R (version 4.1.3; R Foundation, Vienna, Austria). We adjusted for age, sex, hematological events (including neutropenia, leukocytosis, and both neutropenia and leukocytosis in one patient), concomitant psychotropics, and other medications as possible covariates. Continuous variables are presented as means and standard deviations and were compared using the one-way analysis of variance (ANOVA) test. Categorical variables are expressed as numbers and percentages and were compared using the chi-square test. All tests were two-tailed with an alpha value of 0.05. We evaluated neutropenia and leukocytosis by comparing CLO, CLO + VAL, and CLO + Li using Fisher’s exact test to obtain pairwise results. Among these planned comparisons, alpha is adjusted using modified Holm-Bonferroni procedure. To adjust for covariates that may affect neutropenia and leukocytosis, we conducted multivariate analysis through logistic regression, with backward elimination on a threshold of 0.1. The Cochran–Armitage test for trends was applied to examine whether there was an increasing or decreasing trend of developing neutropenia and leukocytosis among different age groups, valproate dosage, and lithium dosage. To explore whether multicollinearity existed, we measured the Spearman correlation coefficient for all the variables.

## Results

### Demographic and clinical data

A total of 1084 patients were included in our analysis. Among them, 796 (73.4%) patients were diagnosed with schizophrenia, 149 (13.7%) with schizoaffective disorder, 78 (7.2%) with bipolar disorder, and the remaining 61 (5.7%) with other psychiatric disorders (Table 1).

**Table 1:** Major diagnoses of patients receiving clozapine in the present study

	Total (*n* = 1084)
Schizophrenia	796(73.4%)
Schizoaffective disorder	149(13.7%)
Delusional disorder	1(0.1%)
Brief psychotic disorder	1(0.1%)
Unspecified psychotic disorder	13(1.2%)
Substance induced psychotic disorders	2(0.2%)
Psychotic disorder due to other medical condition	14(1.3%)
Bipolar disorder	78(7.2%)
Depressive disorder	12(1.1%)
Unspecified persistent mood disorder	1(0.1%)
Involuntary movements	1(0.1%)
Autism spectrum disorder	10(0.9%)
Dementia	6(0.6%)

Table 2 reveals the patients’ demographic characteristics and medications at discharge. Among the included patients, 539 (49.7%) were men. The average age was 41.06 ± 12.81 (mean ± standard deviation) years and average daily clozapine dosage was 242.08 ± 148.86 mg. Concomitant antipsychotics were prescribed to 325 (30.0%) patients, with 245 (22.6%) receiving FGAs and 86 (7.9%) of them receiving SGAs. Antidepressants were prescribed to 164 (15.1%) patients. Ten (0.9%) patients were prescribed with mood stabilizers other than sodium valproate and lithium. Regarding other concurrent medications, 376 (34.7%) patients in the cohort were taking anticholinergics, 559 (51.6%) were taking benzodiazepine, 378 (34.9%) were taking antihypertensives, 29 (2.7%) were taking antidiabetics, 23 (2.1%) were taking antihyperlipidemics, and 22 (2.0%) were taking antiepileptics (Table 2).

**Table 2:** Demographic characteristics and medications: bivariate analysis

	Total (*n* = 1084)	CLO (n = 700)	CLO + VAL (*n* =319)	CLO + Li (n = 45)	CLO + VAL + Li(n = 20)	P value
**Neutropenia**	48 (4.4%)	20 (2.9%)	28 (8.8%)	0 (0.0%)	0 (0.0%)^b^	**< .001** ^b^
**Leukocytosis**	453 (41.8%)	277 (39.6%)	134 (42.0%)	32 (71.1%)	10 (50.0%)	**< .001** ^b^
**Neutropenia + ** **Leukocytosis**	7 (0.6%)	5 (0.7%)	2 (0.6%)	0 (0.0%)	0 (0.0%)	.924^b^
**Age**	41.06 ± 12.81	42.03 ± 13.32	39.43 ± 11.69	40.63 ± 11.07	34.47 ± 11.71	**.002** ^a^
**Daily clozapine dosage (mg)**	242.08 ± 148.86	236.18 ± 143.02	250.9 ± 155.88	288.89 ± 181.11	202.50 ± 136.36	**.044** ^a^
**Sex**						
Male	539 (49.7%)	310 (44.3%)	194 (60.8%)	24 (53.3%)	11 (55.0%)	**< .001** ^b^
Female	545 (50.3%)	390 (55.7%)	125 (39.2%)	21 (46.7%)	9 (45.0%)	
**Antipsychotics**	325 (30.0%)	193 (27.6%)	115 (36.1%)	13 (28.9%)	4 (20.0%)	**.037** ^b^
First generation antipsychotics	245 (22.6%)	146 (20.9%)	86 (27.0%)	10 (22.2%)	3 (15.0%)	.148^b^
Second generation antipsychotics	86 (7.9%)	52 (7.4%)	30 (9.4%)	3 (6.7%)	1 (5.0%)	.677^b^
**Other mood-** **stabilizers**						
Lamotrigine	8 (0.7%)	6 (0.9%)	1 (0.3%)	1 (2.2%)	0 (0.0%)	.489^b^
Oxcarbazepine	2 (0.2%)	1 (0.1%)	1 (0.3%)	0 (0.0%)	0 (0.0%)	.925^b^
**Antidepressant**	164 (15.1%)	119 (17.0%)	37 (11.6%)	7 (15.6%)	1 (5.0%)	.085^b^
Antidepressant total dosage (DDD)	1.15 ± 0.91	1.13 ± 0.93	1.33 ± 0.85	0.66 ± 0.65	0.5	.252^a^
SSRI	111 (10.2)	79 (11.3)	30 (9.4)	1 (2.2)	1(5.0)	.185^b^
SSRI total dosage (DDD)	1.41 ± 0.88	1.41 ± 0.91	1.44 ± 0.81	2.0	0.5	.676^a^
**Anticholinergics**	376 (34.7)	214 (30.6%)	139 (43.6%)	15 (33.3%)	8 (40.0%)	**.001** ^b^
**Benzodiazepine**	559 (51.6%)	339 (48.4%)	180 (56.4%)	28 (62.2%)	12 (60.0%)	**.039** ^b^
**Antihypertensives**	378 (34.9)	219 (31.3%)	127 (39.8%)	26 (57.8%)	6 (30.0%)	**< .001** ^b^
**Antidiabetics**	29 (2.7%)	17 (2.4%)	11 (3.4%)	1 (2.2%)	0 (0.0%)	.687^b^
**Antihyperlipidemics**	23 (2.1%)	11 (1.6%)	8 (2.5%)	4 (8.9%)	0 (0.0%)	**.009** ^b^
**Antiepileptics**	22 (2.0%)	13 (1.9%)	8 (2.5%)	1 (2.2%)	0 (0.0%)	.827^b^

Bold *p* values indicate a statistically significant difference

a: Continuous variables, displayed as mean ± standard deviation, were tested using one-way ANOVA

b: Categorical variables, displayed as number (percentage), were tested using the chi-square test

### Prevalence of neutropenia, leukocytosis, and related factors

A total of 501 events of neutropenia and leukocytosis occurred among all participants during the follow-up period. Specifically, 2.9% of patients in the CLO group and 8.8% of patients in the CLO + VAL group developed neutropenia, whereas 39.6% of patients in the CLO group, 42% of patients in the CLO + VAL group, 71.1% of patients in the CLO + Li group, and 50% of patients the CLO + VAL + Li group developed leukocytosis. Concerning neutropenia, Fisher’s exact test demonstrated that the CLO + VAL group had a significantly higher ratio of neutropenia (*p* < .001; Table 3). There was no significant difference in the incidence of neutropenia between the group receiving CLO + VAL and the group receiving CLO + Li. In this study, we did not observe any neutropenia event in the CLO + Li group or in the CLO + VAL + Li group. As for leukocytosis, concomitant lithium administration was associated with a higher ratio of leukocytosis event (*p* < .001; Table 3). We also found seven cases (five in the CLO group and two in the CLO + VAL group) who had developed both neutropenia and leukocytosis during the 3-year follow-up. However, the ratios were not significantly different across the four groups (Table 2).

**Table 3:** Neutropenia and leukocytosis: *p* value obtained by Fisher’s exact test (2 × 2 table)

	Neutropenia	Leukocytosis
**CLO vs. CLO + VAL**	**< 0.001**	0.491
**CLO vs. CLO + Li**	0.626	**< 0.001**
**CLO + VAL vs. CLO + Li**	0.035	**< 0.001**

Bold and underlined *p* values indicate a statistically significant difference

Alpha is modified using modified Holm-Bonferroni procedure. The number of planned pairwise comparison is three for neutropenia and leukocytosis each; therefore, the p value of CLO + VAL vs. CLO + Li is considered to be insignificant (0.035 > 0.05/2) because this value is the second smallest across the three comparisons in neutropenia

### Result of logistic regression

After adjustment for covariates (i.e., age, sex, hematological events, daily dosage of clozapine, concomitant psychotropics, and other medications) through logistic regression, the CLO + VAL group (odds ratio [OR] = 3.49, *p* < .001, compared with the CLO group) and age (OR = 1.03, *p* = .007) were significantly associated with an increased risk of neutropenia. The CLO + Li group (OR = 3.39, *p* < .001, compared with the CLO group), younger age (OR = 0.97, *p* < .001), clozapine daily dosage (OR = 1.01, *p* = .025), and benzodiazepine use (OR = 1.64, *p* < .001) were significantly associated with leukocytosis development (Table 4).

**Table 4:** Multivariate logistic regression analysis of neutropenia and leukocytosis (final model after variable selection of backward elimination)

	Neutropenia			Leukocytosis		
	Odds ratio	95%CI	P value	Odds ratio	95%CI	P value
**CLO group**	1			1		
CLO + VAL group	3.49	1.91–6.39	**< 0.001**	0.92	0.69–1.22	0.548
CLO + Li group	NA^a^	NA^a^	NA^a^	3.39	1.72–6.68	**< 0.001**
CLO +VAL + Li group	NA^a^	NA^a^	NA^a^	1.14	0.46–2.83	0.781
**Age**	1.03	1.01–1.06	**0.007**	0.97	0.95–0.98	**< 0.001**
**Sex** ^b^				1.39	1.08–1.79	**.012** ^b^
**Clozapine daily ** **dosage** ^**c**^				1.11	1.02–1.22	**0.015**
**Benzodiazepine**				1.64	1.27–2.13	**< 0.001**

Bold and underlined *p* values indicate a statistically significant difference

Values obtained using logistic regression, with backward elimination and a threshold of 0.1. Variables used in the first step of the logistic regression were age, clozapine dosage, sex, antipsychotics use, antidepressants use, anticholinergics use, benzodiazepine use, antihypertensives use, antidiabetics use, antihyperlipidemics use, and antiepileptics use. Omnibus Tests of Model Coefficients revealed statistical significance

a: No patient reported to have neutropenia when using clozapine and lithium concomitantly; b: Reference was set at female; c: Clozapine daily dosage: at increasing intervals of 100 milligrams per day

In addition, in the two formulas presented in Table 3, individual multicollinearity diagnostics revealed that all variables involved in the first step of logistic regression were nonsignificant due to multicollinearity, meaning that every variable had a variance inflation factor (VIF) < 1.5.

### Trend for neutropenia and leukocytosis according to age, dosage of valproate, and dosage of lithium

**Fig. 1:**
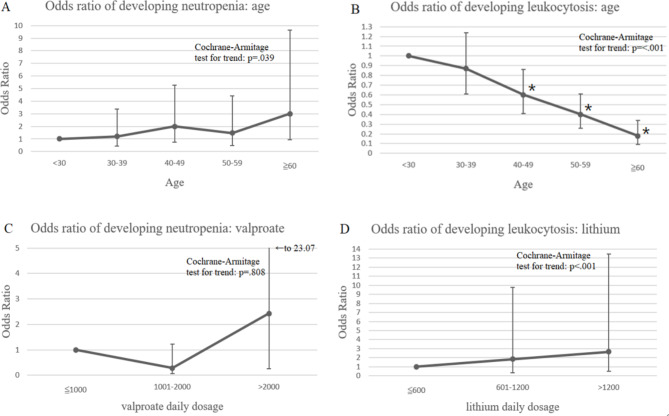
Linear trend for neutropenia and leukocytosis, stratified by age, valproate use, and lithium use

To explore whether there was an increasing or decreasing trend of developing neutropenia and leukocytosis according to age, dosage of valproate, and dosage of lithium, we conducted the Cochran–Armitage test, wherein, individuals were stratified by age (i.e., aged < 30, aged 30–39, aged 40–49, aged 50–59, aged ≥ 60 years). With the group of < 30 years as the reference, neutropenia development did not differ significantly among the subgroups. However, with an increase in age, a trend of increased neutropenic events was observed (*p* = .039; Fig. 1A). In addition, to examine the relationship between valproate daily dosage and neutropenia, we classified the valproate dosage into three subgroups (i.e., dosage ≤ 1000 mg, 1001–2000 mg, and > 2000 mg), with dosage ≤ 1000 mg serving as the reference group. We found no significant difference in each subgroup nor a trend of decreasing or increasing incidence of neutropenia with an increase in valproate dosage (*p* = .808; Fig. 1C).

Regarding leukocytosis, compared with the reference group (< 30 years), those aged 40–49, 50–59, and ≥ 60 had significantly lower risks of developing leukocytosis. Moreover, a decreasing trend of leukocytosis development was observed with increasing age (*p* < .001; Fig. 1B). Regarding lithium dosage, with a dosage of < 600 mg as the reference group, each of the other two subgroups (i.e., 601–1200 mg and > 1200 mg) demonstrated no significant difference in leukocytosis development; however, an increasing trend of leukocytosis incidence with an increase in lithium daily dosage was observed (*p* < .001; Fig. 1D).

Adjusted variables include clozapine dosage, sex, antipsychotics use, antidepressants use, anticholinergics use, benzodiazepine use, antihypertensives use, antidiabetics use, antihyperlipidemics use, and antiepileptics use.

### Relationship between the covariates

The Spearman correlation matrix in Supplementary Fig. 2. reveals that the correlations between many covariates were nonsignificant, and a few covariates were significantly but weakly correlated, as evidenced by the correlation coefficients, which were all less than 0.3. Therefore, the correlation between the covariates in our analysis may not affect our main outcome.

## Discussion

Our study found a significantly increased risk of valproate-related neutropenia and lithium-related leukocytosis in patients receiving clozapine. These findings were independent of other variables, such as age, sex, daily dose of clozapine, and other concomitant medications. Younger age, male sex, and concurrent benzodiazepine use might be linked to leukocytosis, and older age is a risk factor for neutropenia. Lithium dosage increase is related to an upward trend of leukocytosis development, but such a trend was not noted between valproate dosage increase and neutropenia development.

On the basis of our literature review, we enrolled a relatively large number (*N* = 1084) of patients compared with other studies on valproate-related neutropenia and lithium-related leukocytosis. Our research data were from one large psychiatric institution, and the data were collected between 2006 and 2020.

We observed that valproate coadministration is a significant risk factor (OR = 3.49) for the development of clozapine-associated neutropenia. This finding was consistent with those of other studies [16, 26, 27]. Clozapine by itself would induce neutropenia; however, the precise underlying mechanisms remain unclear. One popular hypothesis is that the bioactivation of clozapine produces a chemically reactive nitrenium ion, which causes apoptosis to neutrophils as well as toxicity to stromal cells, the precursors of neutrophils in bone marrow [28]. Clozapine is metabolized by CYP1A2 and CYP3A4, with CYP1A2 playing a major role [26–28]. Clozapine metabolism through CYP1A2 is competitively inhibited by valproate [29]. Additionally, valproate by itself may induce blood dyscrasias by affecting the differentiation of normal multipotent hematopoietic progenitors. The risk of blood dyscrasia is higher when valproate is used in combination with clozapine [30]. In contrast to the mentioned UK study [16], we did not find a dose-dependent effect of sodium valproate on neutropenia development.

In the present study, another mood stabilizer, lithium, increased the risk of leukocytosis in patients treated with clozapine (OR = 3.39). The precise mechanism by which lithium increases WBC count remains unclear. Putative theories include an increase in granulocyte production, direct stem-cell stimulation, cytokine stimulation, redistribution of demarginated leukocytes, and increased cortisol production [31]. Lithium has been used to treat clozapine-induced neutropenia [21, 32] and to prevent neutropenia during clozapine rechallenge [33], with a minimum serum lithium level of 0.4 mmol/L required for such effects [34]. Nevertheless, prescribing clozapine to a patient already under lithium may result in leukocytosis [35]. Interestingly, although leukocytosis appears unrelated to the lithium plasma level [27], we observed a trend of increasing leukocytosis with increased daily lithium dosage.

Patients did not tend to present higher leukocytosis nor neutropenia events regarding coadministration use of clozapine and both valproate and lithium. Our cohort found that 10 (50%) of the patients under the two mood stabilizers developed leukocytosis, while none of the patients developed neutropenia. However, the post-hoc analysis after the chi-square test did not show a higher ratio of leukocytosis event. As our sample size of patients under clozapine combined with two mood stabilizers is small, future investigation with larger sample size is needed to explore the incidence of the subsequent hematological events.

Aging is another risk factor for neutropenia in patients under clozapine. Aging is known to cause changes in absorption, distribution, biotransformation, and elimination of drugs [36]. Hepatic clearance of a drug depends on hepatic enzyme activity and blood supply to the liver, which decreases with aging [37, 38]. As aging is associated with an increase in adipose mass, the distribution volume is greater for clozapine, a lipid-soluble drug, resulting in an increase in plasma half-lives [39]. Furthermore, functional decline in the dopaminergic system due to aging is a predictor of low antipsychotic dose prescriptions for older patients [40]. Therefore, clozapine is indicated for older adults only when other antipsychotics have failed [41].

Our data analysis revealed a higher risk of leukocytosis in benzodiazepine-cotreated patients (OR = 1.64). However, current evidence suggests that concomitant benzodiazepines use is a risk factor for transient anemia, but not for leukocytosis [42]. Benzodiazepines exert effects such as sedation and anxiolysis by acting upon different GABA-A receptor subtypes, which is different from the pharmacology of clozapine [43]. Furthermore, strong emotional reactions and illness may induce leukocytosis [44]. In clinical settings, patients may exhibit more emotional reactions such as anxiety or agitation; in such cases, leukocytosis may be related to the emotional behaviors rather than to benzodiazepine use.

Evidence indicates that male sex is a risk factor for leukocytosis in patients taking clozapine [42, 45]. Our study also observed male patients to have higher risks of leukocytosis. Furthermore, younger patients demonstrated a higher risk of leukocytosis. However, reports regarding younger patients developing clozapine-associated leukocytosis are limited. Some remote evidence from stem-cell research has suggested that aging interferes with leukopoiesis [46, 47]. Future studies are warranted to confirm our findings.

A slightly increased risk of leukocytosis was observed with higher clozapine doses (OR = 1.01, 95% confidence interval: 1.00–1.02). Leukocytosis, a generally asymptomatic and benign condition [33], appears to be a potential side effect of clozapine [36]. The mechanisms of clozapine-induced leukocytosis may be related to changes in plasma concentrations of the granulocyte colony-stimulating factor, tumor necrosis factor-α, interleukin (IL)-2 cytokines, and IL-6 cytokines [33]. Moreover, a downward dose titration results in normalization of WBC counts, suggesting a dose-dependent effect of clozapine [48] .

Our study did not replicate a recent finding in the UK demonstrating a reduced risk of neutropenia in patients cotreated with SSRIs [16]. Although the study reported reduced risks of neutropenia in patients treated with SSRIs, a large drug surveillance study with 122,000 psychiatric inpatients [49], which had a similar finding as ours, did not support this finding. In addition, the use of concomitant antidepressants such as mirtazapine and imipramine was associated with neutropenia development. Evidence does not support SSRIs or serotonin–norepinephrine reuptake inhibitors causing neutropenia in patients treated with clozapine [50].

A recent study in Japan involving 3746 patients treated with clozapine reported that 4.9% of the patients developed neutropenia. In our study, the incidence of neutropenia was 2.9%, which was consistent with the 2–3% prevalence described in the literature [5]. Additionally, a recent systematic review reported significantly lower clearance of clozapine in East Asian patients compared with Caucasians [51]. Poor metabolism of clozapine was noted in approximately 10% of Asians; therefore, they needed lower doses to reach therapeutic concentrations [52]. Because of this, Asian patients may be more sensitive to blood dyscrasia (neutropenia or leukocytosis). Data on the concomitant use of clozapine with valproate and lithium in Asian patients are limited. Therefore, a strength of our study is the identification of incidences of neutropenia and leukocytosis between patients treated with clozapine monotherapy alone and subgroups of patients on clozapine cotreated with mood stabilizers.

Our study has some limitations. Because this was a retrospective cohort study, some important predictive factors may have been underestimated. Moreover, because the groups were not randomly assigned, some threats to internal validity may have occurred. Although we analyzed participants’ basic demographic data and common concurrent medications, other factors such as smoking [11], oral contraceptive use [29], obesity [53], and inflammation [54] may have affected the WBC counts. Currently, no clinical registry comprises data on physical conditions and treatments from both psychiatric and general hospitals. Furthermore, because the included patients may have irregular outpatient clinic visits, routine hematologic examination may not be regularly performed. We also did not include the serum levels of both valproate and lithium for our patients at discharge. Additionally, clozapine adherence was not objectively confirmed because monitoring clozapine levels is not the standard practice in Taiwan.

## Conclusion

Our study demonstrated that concomitant administration of valproate with clozapine was significantly associated with the neutropenia three-fold risk increase. Concomitant use of clozapine with lithium was associated with three-fold higher risks of leukocytosis. Therefore, regular follow-up hematology profiles are warranted in patients who received combined treatment with clozapine and valproate/lithium.

## Electronic supplementary material

Below is the link to the electronic supplementary material.

Supplementary Material 1 Supplementary Figures

## Data Availability

The datasets generated and/or analysed during the current study are not publicly available due to the ethical restrictions from the Institutional Review Board, but are available from the corresponding author on reasonable request.
