# Supplementary material for: Valproate-related neutropenia and lithium-related leukocytosis in patients treated with clozapine: a retrospective cohort study
Source: BMC Psychiatry. 2023 Mar 15;23:170. doi: 10.1186/s12888-023-04659-2 (PMC10018892; doi:10.1186/s12888-023-04659-2)
Supplement: Supplementary file 1 — Supplementary Material 1 Supplementary Figures [file 12888_2023_4659_MOESM1_ESM.docx]

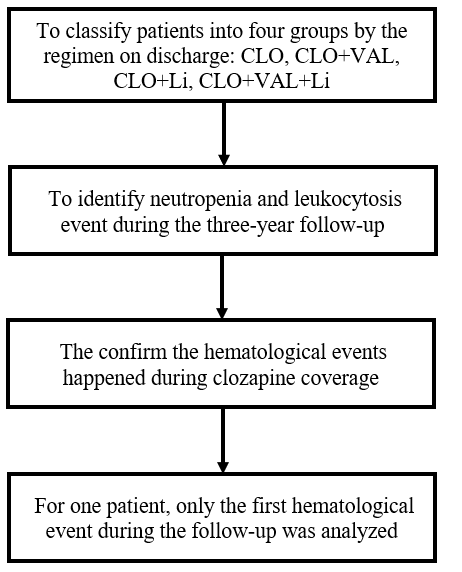


Supplementary Figure 1. Flowchart of patient classification and hematological event identification

Supplementary Figure 2. Spearman correlation matrix of the variables in multivariate analysis


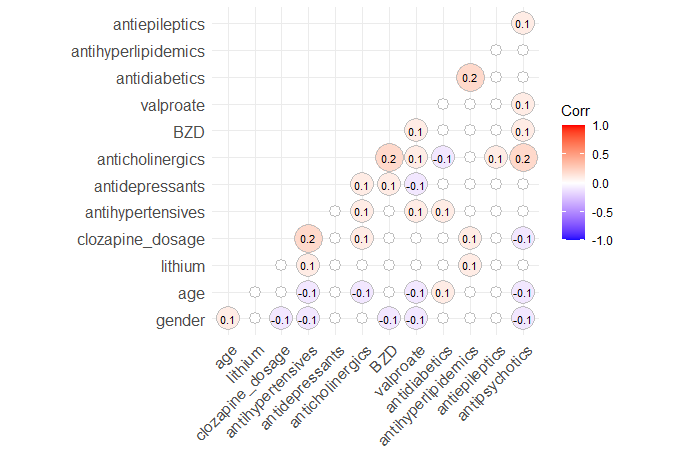


Correlations left blank are nonsignificant.
